# Real‐Time NMR Spectroscopy for Studying Metabolism

**DOI:** 10.1002/anie.201912919

**Published:** 2019-12-20

**Authors:** Islam Alshamleh, Nina Krause, Christian Richter, Nina Kurrle, Hubert Serve, Ulrich L. Günther, Harald Schwalbe

**Affiliations:** ^1^ Institute of Organic Chemistry and Chemical Biology Center for Biomolecular Magnetic Resonance (BMRZ) Johann Wolfgang Goethe-University Frankfurt Max-von-Laue Str. 7 60438 Frankfurt Germany; ^2^ German Cancer Consortium (DKTK) and DKFZ 69120 Heidelberg Germany; ^3^ Department of Medicine 2, Hematology/Oncology Goethe University 60590 Frankfurt am Main Germany; ^4^ Frankfurt Cancer Institute (FCI) 60590 Frankfurt am Main Germany; ^5^ Institute of Chemistry and Metabolomics University of Luebeck Ratzeburger Allee 160 23562 Luebeck Germany

**Keywords:** biological chemistry, cell studies, metabolism, personalized medicine, real-time NMR spectroscopy

## Abstract

Current metabolomics approaches utilize cellular metabolite extracts, are destructive, and require high cell numbers. We introduce here an approach that enables the monitoring of cellular metabolism at lower cell numbers by observing the consumption/production of different metabolites over several kinetic data points of up to 48 hours. Our approach does not influence cellular viability, as we optimized the cellular matrix in comparison to other materials used in a variety of in‐cell NMR spectroscopy experiments. We are able to monitor real‐time metabolism of primary patient cells, which are extremely sensitive to external stress. Measurements are set up in an interleaved manner with short acquisition times (approximately 7 minutes per sample), which allows the monitoring of up to 15 patient samples simultaneously. Further, we implemented our approach for performing tracer‐based assays. Our approach will be important not only in the metabolomics fields, but also in individualized diagnostics.

## Graphical abstract

**Seeing is believing**: A newly developed approach for monitoring living‐cell metabolism in a cell‐friendly environment is reported, paving the way for bringing NMR spectroscopy closer to personalized medicine.

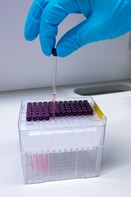

Over the last decade, metabolomics, the study of cellular metabolism, has become increasingly important. Metabolomic studies address how cells fulfil their energy needs: metabolic pathways for energy production are elucidated by quantification of metabolite concentration. Modes of metabolic rewiring that cells undergo to overcome nutrient deprivation and cellular stress can be detected.

Recently, it has been shown that changes in metabolism are a vulnerability that can be targeted in cancer cells (reviewed in ref. 1, 2). In fact, the metabolism of malignant cells is different from healthy cells as these cells reprogram their metabolic pathways to fulfil the high energy demands of highly proliferating cells and to develop resistance to drug treatment.^3^, ^4^ Metabolism targeting is becoming a core research area in therapeutics development for different cancers, including acute myeloid leukemia (AML), a hematological malignancy that results in uncontrolled cellular proliferation.^5^ In fact, several inhibitors of metabolism are currently being evaluated in clinical trials (l‐asparaginase and CPI‐613)^4^, ^6^, ^7^, ^8^ and some others have already been approved for AML treatment (Venetoclax and isocitrate dehydrogenase (IDH) inhibitors).^9^, ^10^

Nuclear magnetic resonance (NMR) spectroscopy and mass spectrometry are prime technologies to phenotype the metabolism of different cancer cell types. NMR spectroscopy provides remarkably reproducible results, great ease of sample preparation, and the possibility of preserving samples over extended periods of time.^11^ Using 1D and 2D isotope‐filtered experiments, different metabolic pathways can be simultaneously tracked when using isotope‐labeled precursor metabolites.^12^ Currently, NMR metabolomics samples are prepared by harvesting cells, extracting their metabolic content, and quantifying the change in their concentration.^13^ However, as metabolism is a highly dynamic process, the concentrations can change rapidly over time which makes it difficult and labor‐intensive to make metabolite extracts at different time points to accurately assign metabolic fluctuations over a time course.

Another layer of complexity is added when investigating metabolic profiles under different conditions (for example, adaption to hypoxic conditions), where one needs to differentiate between acute metabolic response, adaptations, and chronic rewiring in the cells. Up‐to‐now, such studies require high cell numbers (approximately 1×10^7^ cells)^14^ for NMR spectroscopic analysis, which are often difficult to obtain when studying primary patient cells, making NMR spectroscopy unattractive for this kind of samples. Moreover, materials used for sample preparation, in particular agarose gels in previously described methods for monitoring live‐cell metabolism,^15^, ^16^, ^17^, ^18^ can be cell‐unfriendly, can further lead to reduced metabolite diffusion rates and induce environmental stress that obscures the real metabolic fingerprint of the cell.^17^ Such agarose preparations, however, are commonly used also for in‐cell NMR spectroscopy, although it may compromise cell viability.^19^, ^20^

To address these challenges, we introduce an automated real‐time NMR spectroscopy approach, which enables live monitoring of metabolism changes in viable AML cells. The newly developed method allowed us to monitor the metabolism of primary patient cells in an automated fashion, extending this method to individualized diagnostics required for personalized medicine approaches. In principle, our method allows for a simultaneous interleaved measurement of several patient samples (10–15 samples), due to the short NMR measurement time of 7 minutes. For ethical reasons, we demonstrate this experimental schedule, however, not on different primary patient samples but apply the acquisition scheme to primary cells from a single patient.

Different to previous experimental designs,^13^ the newly developed approach is not destructive, since cells are preserved and used again for other experiments or diagnostic procedures (low TMSP (trimethylsilylpropanoic acid) and D_2_O concentrations are reported to be non‐toxic).^21^, ^22^ Furthermore, it needs a small number of cells (approximately 5×10^5^ cells or even fewer) compared to (approximately 1×10^7^ cells) required for current metabolites extraction settings.

A sample changer supplemented with temperature control typically set to 37 °C and a robot that alternates the samples without temperature change into the spectrometer has been used (Figure 1 A). Several spectra are recorded over time to detect changes in the uptake and efflux of the individual metabolites (Figure 1 B). To prevent cell sedimentation in the NMR tube, we optimized our approach by preparing samples in a cell culture media with a cell‐friendly matrix. We first investigated the impact of agarose, a widely used material for NMR metabolomics and in‐cell experiments. We observed a significant impact on cellular ATP levels (a measure of viability, Figure 2 A). To overcome this, we replaced agarose by 40 % methylcellulose media as a matrix. Methylcellulose medium is usually used for assays of highly sensitive cells, including stem cells assays. Indeed, methylcellulose did not influence the cellular viability or metabolism (Figure 2 B). In fact, a concurrently emerging report has shown the advantages of using methylcellulose for studying protein interactions in living cells.^23^ The reliability of the approach was further validated by investigating the cellular responses to interferences with a certain signalling/survival pathway known to influence cellular metabolism. Internal tandem repeats (ITDs) in the fms‐like tyrosine kinase (FLT3‐ITD, commonly mutated in AML^5^) are reported to induce high glucose uptake. FLT3 inhibition leads to reduction in glucose consumption and hence, reduced glycolytic activity.^24^, ^25^ Midostaurin, an FDA‐approved FLT3 inhibitor,^26^ was used and its effects on metabolism were evaluated using the presented approach. The FLT3‐ITD positive cell line MOLM‐13 showed the expected drug‐induced metabolic shifts of reduction in glucose uptake (higher retention of glucose in the media) in the midostaurin group (Figure 2 C).

**Figure 1:**
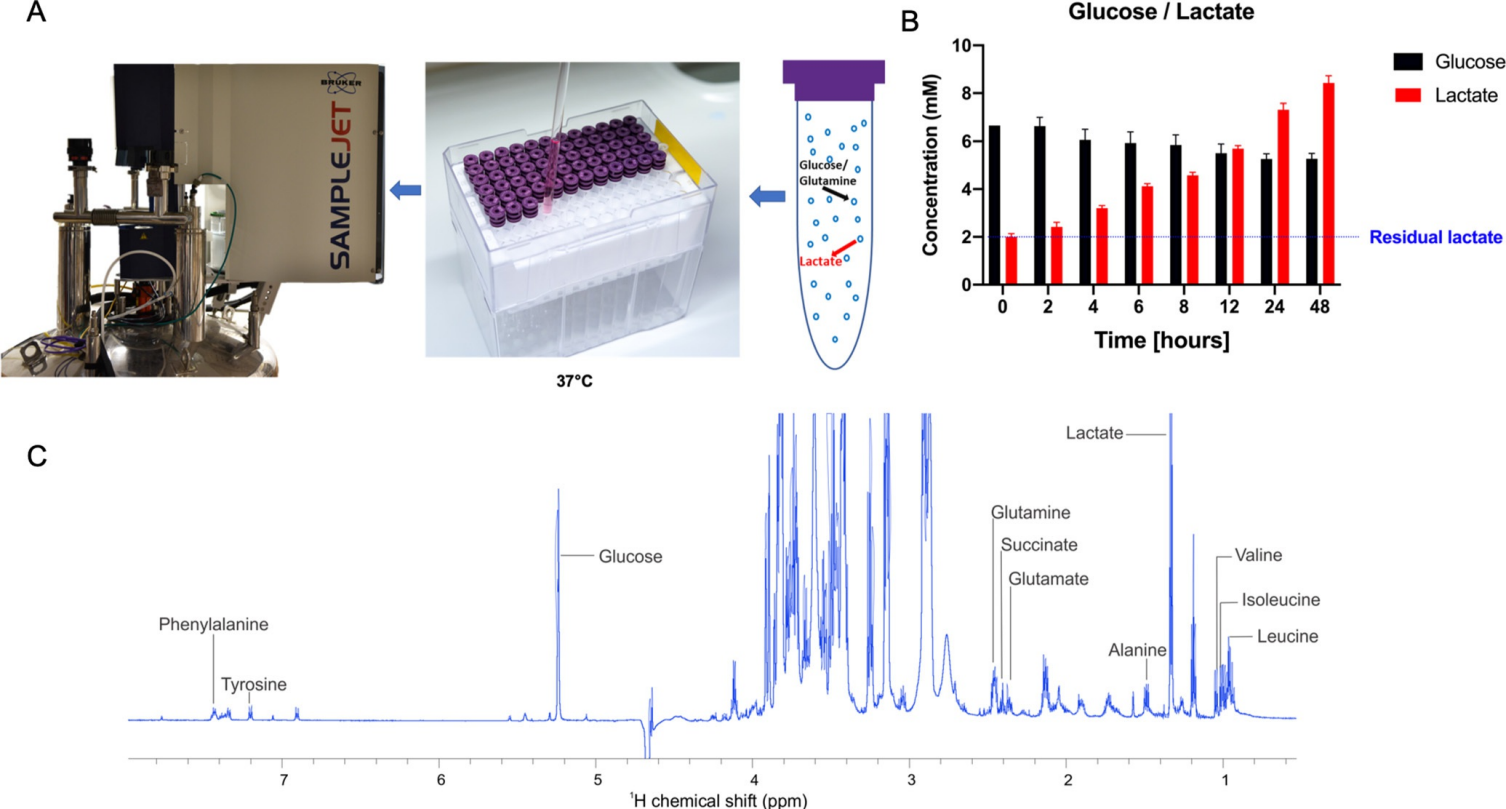
A) Graphical illustration of sample and experimental setup in real‐time NMR spectroscopy. B) Metabolic changes in MOLM‐13 cells represented by glucose uptake and lactate production over a period of 48 hours. C) Peak assignments used for metabolite quantification.

**Figure 2:**
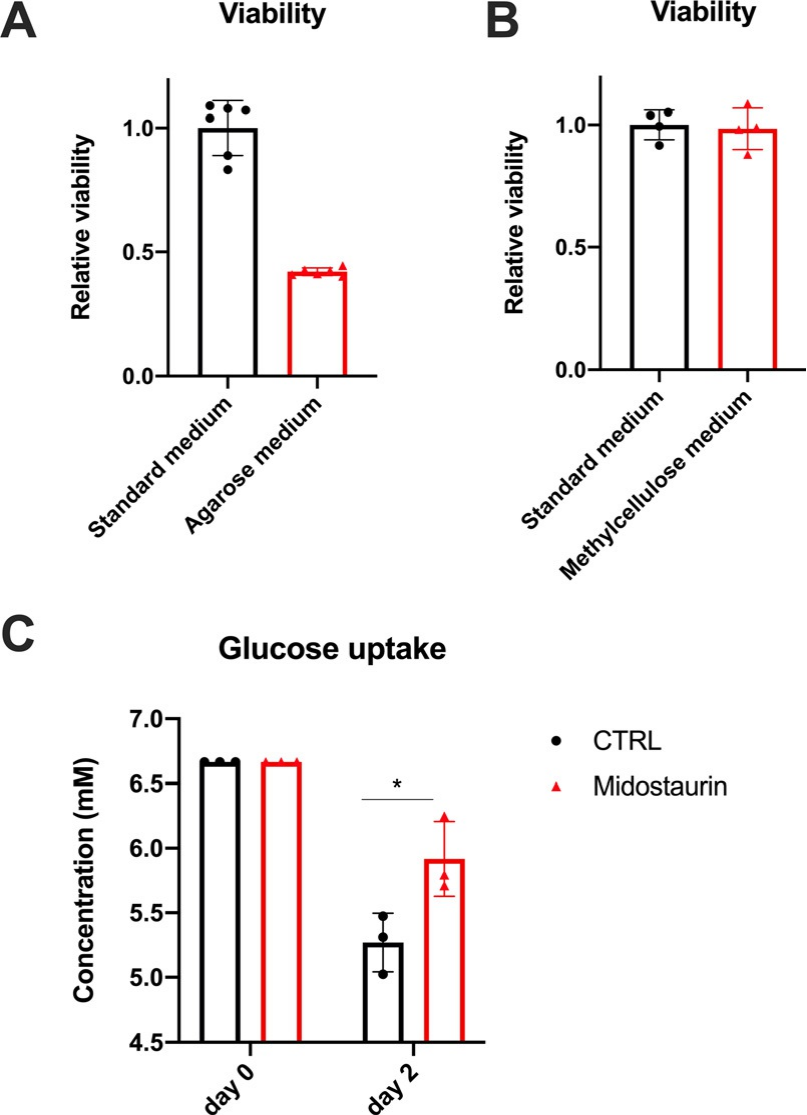
ATP levels (indicating cellular viability) of MOLM‐13 cells grown in standard RPMI medium vs. A) agarose medium and B) methylcellulose medium. C) Glucose consumption upon treatment with 30 nm midostaurin (approximately IC_50_).

To measure O_2_ and pH levels in the NMR tube, we performed measurements of both 48 hours after plating the cells. Oxygen levels were between 1.4 % and 3.2 %. Physiologically, 1–6 % oxygen is usually the normal oxygen level encountered by the leukemic cells in the bone marrow, depending on the distance from the endosteal niche.^27^, ^28^ Hence, our technique reflects metabolic measurements of physiological‐like conditions. The pH value was around 7.4, 0.2 pH units below that of the standard cell culture conditions (pH 7.6), thus, within the normal range of blood pH.^29^

Moreover, this approach was successfully applied to study mononuclear cells isolated from bone marrow aspirates of an AML patient. Typically, AML patient cells are highly sensitive to culture conditions. Once put in the methylcellulose conditions within the NMR sample tube, cells behaved completely normal in terms of their metabolic activity. They consumed glucose and produced lactate, indicating active glucose energy metabolism (Figure 3). Furthermore, they consumed glutamine indicating energy and redox metabolism, branched chain and other amino acids as building blocks for protein biosynthesis, energy production and/ or DNA methylation activity.

**Figure 3:**
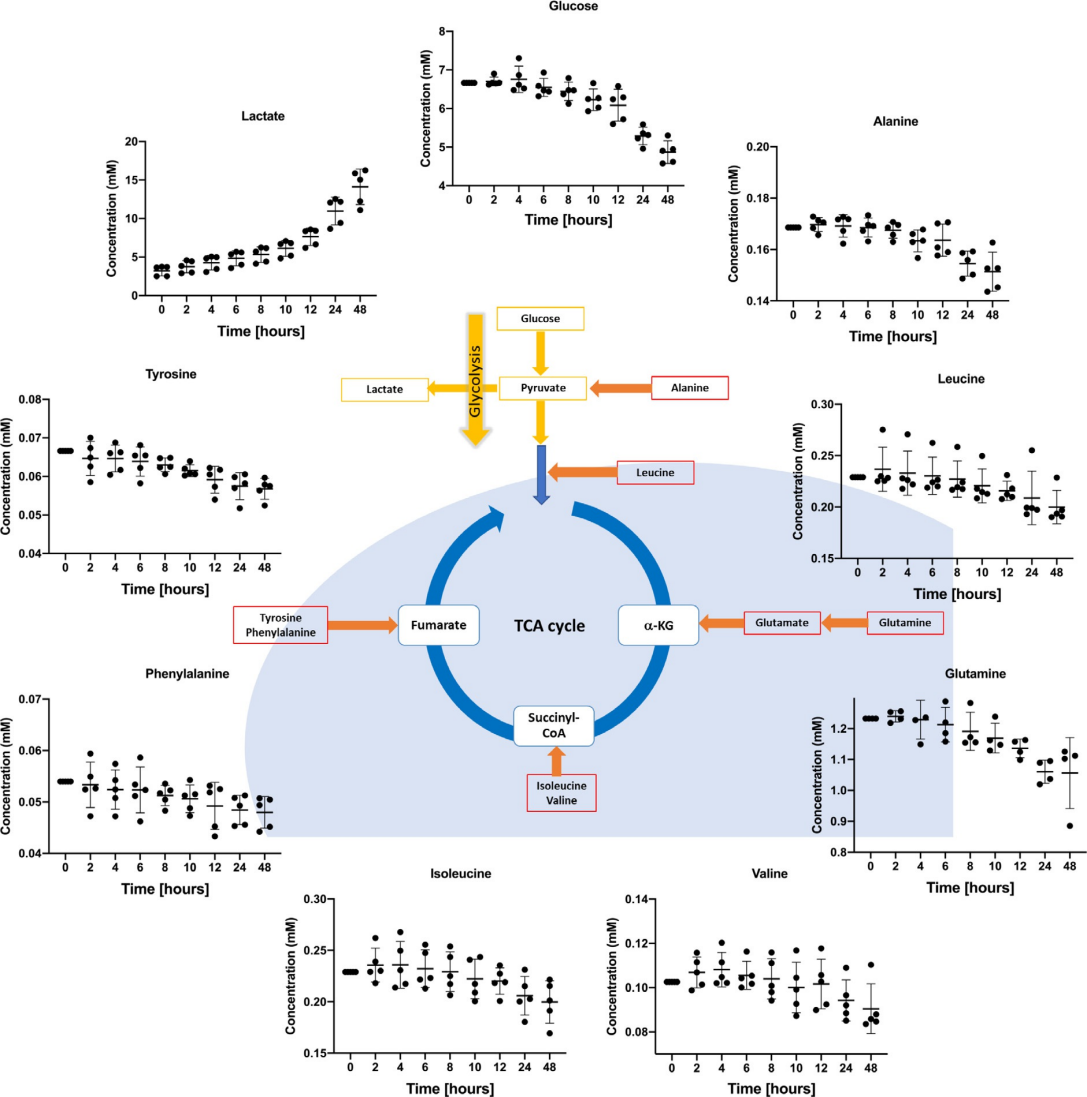
Real‐time flux of different metabolites in mononuclear cells isolated from bone marrow aspirates of an acute myeloid leukemia patient (prepared with 5 technical replicates). It shows how glucose and other amino acids are taken up and, on the other hand, how some energy metabolites are being produced and excreted by the cell.

Finally, we extended the application of our method to perform tracer‐based assays. Since traditional HSQC experiments are time consuming, which undermines the real‐time characteristics of this approach, a pseudo‐2D experiment was implemented. Utilizing a double filtering approach allows the differentiation between protons attached to ^12^C or ^13^C, as described.^30^ Upon labeling with U‐^13^C‐glucose, an increased ^13^C‐glucose ratio in the cells was observed (Figure 4). Quantification of the changes of glucose concentration was subsequently translated in an increased label incorporation in lactate and alanine.

**Figure 4:**
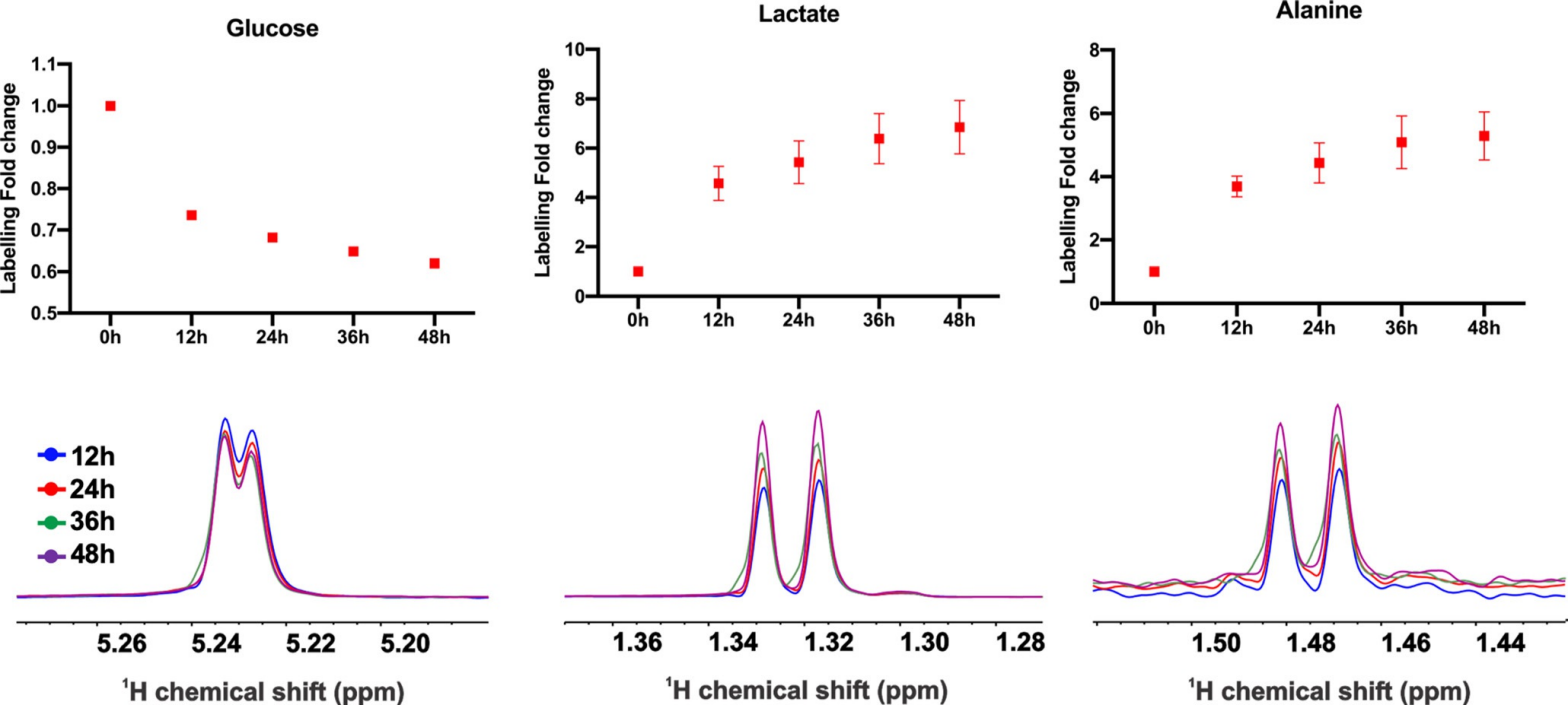
^13^C‐label incorporation in the different metabolites. Labeling in glucose is declining, indicating its consumption while lactate and alanine are being produced. N=4. NMR spectra (from one replicate) shown for the individual metabolites are referenced to glucose peak (scaling is different between the different metabolites).

Here, we introduce a cell‐friendly approach that facilitates the studying of cellular metabolism in a real‐time manner. We demonstrated that this approach does not affect the cellular viability and we could successfully use it to study extremely sensitive cells, such as primary AML cells, thus bringing NMR spectroscopy from the bench closer to the bedside. Moreover, we demonstrated that cells are responsive to any expected changes due to small molecule inhibitors or external stimuli. Finally, we implemented ^13^C filter experiments where we could assign in a tracer‐based setting the label incorporation into the downstream metabolic pathways.

## Conflict of interest

The authors declare no conflict of interest.
